# Transcriptome profiling based on Illumina- and SMRT-based RNA-seq reveals circadian regulation of key pathways in flower bud development in walnut

**DOI:** 10.1371/journal.pone.0260017

**Published:** 2021-11-18

**Authors:** Kai Ma, Xiang Luo, Liqun Han, Yu Zhao, Aisajan Mamat, Ning Li, Chuang Mei, Peng Yan, Rui Zhang, Jianfang Hu, Jixun Wang

**Affiliations:** 1 College of Horticulture, China Agricultural University, Beijing, China; 2 Institute of Horticultural and Crops, Xinjiang Academy of Agricultural Sciences, Urumqi, China; 3 State Key Laboratory of Crop Stress Adaption and Improvement, Henan University, Kaifeng, China; 4 Xinjiang Production and Construction Corps Key Laboratory of Protection and Utilization of Biological Resources in Tarim Basin, Tarim University, Alaer, China; USDA-ARS Southern Regional Research Center, UNITED STATES

## Abstract

Flower bud development is a defining feature of walnut, which contributes to the kernel yield, yield stability, fruit quality and commodity value. However, little is known about the mechanism of the flower bud development in walnut. Here, the stages of walnut female flower bud development were divided into five period (P01-05) by using histological observation. They were further studied through PacBio Iso-Seq and RNA-seq analysis. Accordingly, we obtained 52,875 full-length transcripts, where 4,579 were new transcripts, 3,065 were novel genes, 1,437 were consensus lncRNAs and 20,813 were alternatively spliced isoforms. These transcripts greatly improved the current genome annotation and enhanced our understanding of the walnut transcriptome. Next, RNA sequencing of female flower buds at five periods revealed that circadian rhythm-plant was commonly enriched along with the flower bud developmental gradient. A total of 14 differentially expressed genes (DEGs) were identified, and six of them were confirmed by real-time quantitative analysis. Additionally, six and two differentially expressed clock genes were detected to be regulated by AS events and lncRNAs, respectively. All these detected plant circadian genes form a complex interconnected network to regulate the flower bud development. Thus, investigation of key genes associated with the circadian clock could clarify the process of flower bud development in walnut.

## Introduction

*Juglans regia* L., also known as common walnut, is widely cultivated in temperate regions extending from North, Central and South America to Europe and North Africa because of its high nutritional values [1,2]. Walnut has more than 7000 years of evolutionary and domestication history in China [3], where produced numerous walnut cultivars with elite traits such as early fruiting, high fruit yield, high oil content, thin kernel and so on. Therein, early fruiting, fruit yield and kernel quantity are directly influenced by the intensity and quality of flower bud development. However, the molecular mechanism of flower bud development has remained unclear in walnut.

Flower development is a highly complex physiological, biochemical and morphogenetic process in plant. It is affected by various factors such as, photoperiod [4], circadian clocks [5], hormones [4,6,7], sucrose distribution [8–10] and other metabolic processes [7,11]. However, the advances in the studies of flowering pathway are relatively slow in woody trees. Zhu [11] demonstrated that genes related to oxidoreductase activity and phytohormone metabolism regulate the dormant flower buds of Chinese cherry (*Prunus pseudocerasus*). Muhammad [7] indicated that genes involved to the secondary metabolites biosynthesis and plant hormone signaling affect the bud dormancy in grape. In walnut, the treatment of heat orgibberellic acid (GA_3_) can affect the flowering pattern [12,13]. MicroRNAs sequencing revealed that the differentially expressed miRNAs mainly enriched to ubiquitin mediated proteolysis, RNA degradation and various carbohydrate metabolism pathways were related to the female flower induction [14]. Transcriptome profiling identified 374 genes, which differentially expressed to regulate the transition between female buds and leaf buds [15]. To date, however, none of the reported miRNAs or genes can completely explain the genetic basis of flower bud development in walnut.

The published ‘Chandler’ walnut genome provides an important tool to facilitate the genetic dissection of complex traits [16]. Nevertheless, the gene annotation was built based on the transcriptional data with short reads generated by second-generation sequencing (SGS) [17], which is difficult in dealing with the repeat regions. Additionally, cultivars usually exhibit a strong reduction in genetic variation due to population bottlenecks and strong artificial selection during domestication, implying the limitation of single genome reference based on short-read sequencing. Fortunately, long-read or full-length cDNA sequences based on a third-generation sequencing (TGS) platform can perfectly overcome the problems of SGS platforms [18,19] and correct the gene annotations [20,21]. Furthermore, full-length cDNA sequences are particularly helpful to analyze different transcript isoforms generated by alternate splicing and non-coding RNA (ncRNA).

In this study, we preformed full-length cDNA and transcriptome sequencing from female flower buds, and analyses the differentially expressed genes (DEGs), alternate splicing (AS) and long non-coding RNA (lncRNA) related to flower bud development in walnut. Our results provided valuable resources for elucidating the development of the flower buds in walnut.

## Materials and methods

### Plant materials

‘Wen 185’, one of the most widely cultivated walnut varieties in Xinjiang, was grown under natural conditions in the Xinjiang Fruit Science Experiment Station of Ministry of Agriculture and Rural Affairs, Yecheng, China. Female flower buds were collected from the upper bud of the axillary bud in the 2-3^rd^ position below the top bud of terminal. They were collected five stages (P01, P02, P03, P04 and P05) in an interval of 20 days starting from the initial period of flowering (P01) on 5^th^ April 2018 to 25^th^ June 2018. A total of 18 female flower buds were collected for each stage. Among them, 15 buds were stored in -80°C, and the others were fixed in flavone acetic acid (FAA) fixative after peeling off the outer scales before use.

### Histological observation using paraffin sections

The fixed female flower buds at different stages were used for histological observation using paraffin sections. These buds were dehydrated with a continuous gradient of (%) ethanol and were embedded in paraffin. Samples were cut into 8–10μm slices (Leica Microtome, Germany), deparaffinized with xylene, and hydrated in a decreasing ethanol series. The sections were stained with Safranin and Fast Green and mounted with neutral gum. Finally, the slices were observed under a Motic microscope (Motic AE31, China).

### RNA preparation

Each sample was pooled with five buds from the same walnut tree, and three biological repeats (from three different walnut trees) were performed for five different stages. The total RNA of 15 samples were extracted individually by using RNA preparation kit (Tiangen Biotech, Beijing, China) following the provided protocol. RNA concentration was measured using NanoDrop 2000 (Thermo Scientific, Waltham, MA, USA). RNA integrity was assessed using the RNA Nano 6000 Assay Kit of the Agilent Bioanalyzer 2100 system (Agilent Technologies, CA, USA). Then, quantified RNA samples were used for constructing cDNA libraries subsequently.

### PacBio cDNA library construction and third-generation sequencing

One mixed RNA of all 15 samples in five periods was used to construct PacBio cDNA library. The sequencing library was prepared according to the Iso-Seq protocol as described by Pacific Biosciences (P/N100-377-100-05 and P/N100-377-100-04). The SMARTer PCR cDNA Synthesis Kit was used to synthesize cDNA from the same RNA samples used for PacBio sequencing. Size fractionation and selection (1-2kb, 2-3kb, and 3-6kb) were performed using the BluePippin™ Size Selection System (Sage Science, Beverly, MA, USA). The amplified cDNA products were used to generate SMRTbell Template libraries according to the Iso-Seq protocol. Libraries were prepared for sequencing by annealing a sequencing primer and adding polymerase to the primer annealed template. The polymerase-bound template was bound to MagBeads, and sequencing was performed on a PacBio RSII using the provided protocol.

### Quality filtering and error correction of PacBio long reads

TGS subreads were filtered using the standard protocols in the SMRT Analysis software suite (http://www.pacificbiosciences.com), and reads of insert (ROIs) were obtained. After examining for poly (A) signal and 5’ and 3’ adaptors, full-length and non-full-length cDNA reads were recognized. Consensus isoforms were identified using the algorithm of iterative clustering for error correction and further polished to obtain high-quality consensus isoforms. The raw Illumina SGS reads were filtered to remove adaptor sequences, ambiguous reads with ’N’ bases, and low-quality reads. Afterward, error correction of low-quality isoforms was conducted using the SGS reads with the software proovread 2.13.841. Redundant isoforms were then removed to generate a high-quality transcript dataset for walnut using the program CD-HIT 4.6.142 (http://weizhongli-lab.org/cd-hit/).

### Functional annotation of transcripts

Functional annotations were conducted by using BLASTX (cutoff E-value ≤ 1e-5) against different protein and nucleotide databases of Clusters of Orthologous Groups (COG), Gene Ontology (GO), Kyoto Encyclopedia of Genes and Genomes (KEGG). For each transcript in each database searching, the functional information of the best matched sequence was assigned to the query transcript.

### Predictions of ORF, gene fusion and long non-coding RNAs

To predict the open reading frames (ORFs) in transcripts, we used the package TransDecoder v2.0.1 (https://transdecoder.github.io/) to define putative coding sequences (CDS). The fusion transcript was identified according to the following criterions: 1) A single transcript must map onto the reference genome with two or more loci; 2) The minimum coverage for each loci was 5% length of the total transcript; 3) Total coverage was ≥ 95%; 4) the distance between adjacent loci was at least 10kb. The predicted CDS was searched and confirmed by BLASTX (E-value ≤1e-5) against the protein databases of NR, SWISS-PROT, and KEGG. Those transcripts containing complete ORFs as well as 5’- and 3’-UTR (untranslated regions) were designated as full-length transcripts. To predict long non-coding RNAs (lncRNAs), we used PLEK (predictor of long non-coding RNAs and messenger RNAs based on an improved k-mer scheme (https://sourceforge.net/projects/plek).

### Illumina cDNA library construction and second-generation sequencing

A total of 15 Three RNA samples from five buds in each period were used for Illumina cDNA library construction using NEBNext® Ultra™ RNA Library Prep Kit for Illumina® (NEB, Beverly, MA, USA), following the manufacturer’s recommendations. Qualified libraries were applied to NGS using an Illumina Hiseq 2500 (Illumina, San Diego, CA, USA) to generate 125bp paired-end sequence reads (2×125bp). High-throughput sequencing reported in this study was performed in the Biomarker Technology Co., Ltd (Beijing, China).

### Illumina data analysis

Raw data (raw reads) in fastq format were first processed using in-house perl scripts. In this step, clean data (clean reads) were obtained by removing reads containing adapters, reads containing poly “N” and low-quality reads. These clean reads were then mapped to the reference genome sequence using Tophat2 [22]. Only reads with a perfect match or one mismatch were further analyzed and annotated based on the reference genome. Gene expression levels were estimated by FPKM. Differential expression analysis between two stages was performed using the DESeq package [23]. The resulting *P* values were adjusted using the Benjamini and Hochberg’s approach for controlling the false discovery rate [24]. Genes identified by DESeq with FDR ≤ 0.01 and FC ≥ 2 were defined as differentially expressed. K-means clustering was conducted based on the *Pearson* correlation of gene expression profiles [25].

### GO and KEGG enrichment analysis

Functional annotations of the DEGs were performed and searched against the GO (Gene Ontology) and KEGG (Kyoto Encyclopedia of Genes and Genomics) databases. Classification and enrichment of DEGs were carried out by WEGO and ArigGO [26]. respectively. Metabolic pathway assignments were carried out based on the KEGG orthology database (http://www.genome.ad.jp/kegg/) using KOBAS2.0 [27,28]. GO and KEGG pathway enrichment analysis was conducted by Fisher’s exact test corrected with FDR of 5%.

### Arabidopsis homologous genes searches and phylogenetic analysis of the clock genes

DEGs were used as queries in translated nucleotide BLAST (BLASTX) searches against *Arabidopsis* gene sequences in the *Arabidopsis* Information Resource 10 database (http://www.arabidopsis.org/) to obtain the closest *Arabidopsis* homolog (E-value ≤ e-10). The domains and functional sites in their corresponding proteins were examined with InterProScan [29]. The ClustalW software was used for the sequence alignment between walnut and *Arabidopsis*, and phylogenetic trees were constructed using the MEGA 7.0 software. The neighbor-joining method was performed and 1000 bootstrap test replicates were used during the construction [30].

### Quantitative real-time PCR

RNA samples were isolated from female flower buds in different developmental stages from ‘Wen 185’. qRT-PCR primers were designed with PRIMER PREMIER 6 (PREMIER Biosoft, http://www.premierbiosoft.com/primerdesign/). PCRs were conducted using the StepOnePlus Real-Time PCR System (Applied Biosystems, Foster City, CA, USA). Data were analysed using the 2^−ΔΔCt^ method to calculate relative gene expression [31]. S1 Table lists all primers used in the qRT-PCR experiments.

## Results

### Morphological characteristics of walnut floral development

**Fig 1:**
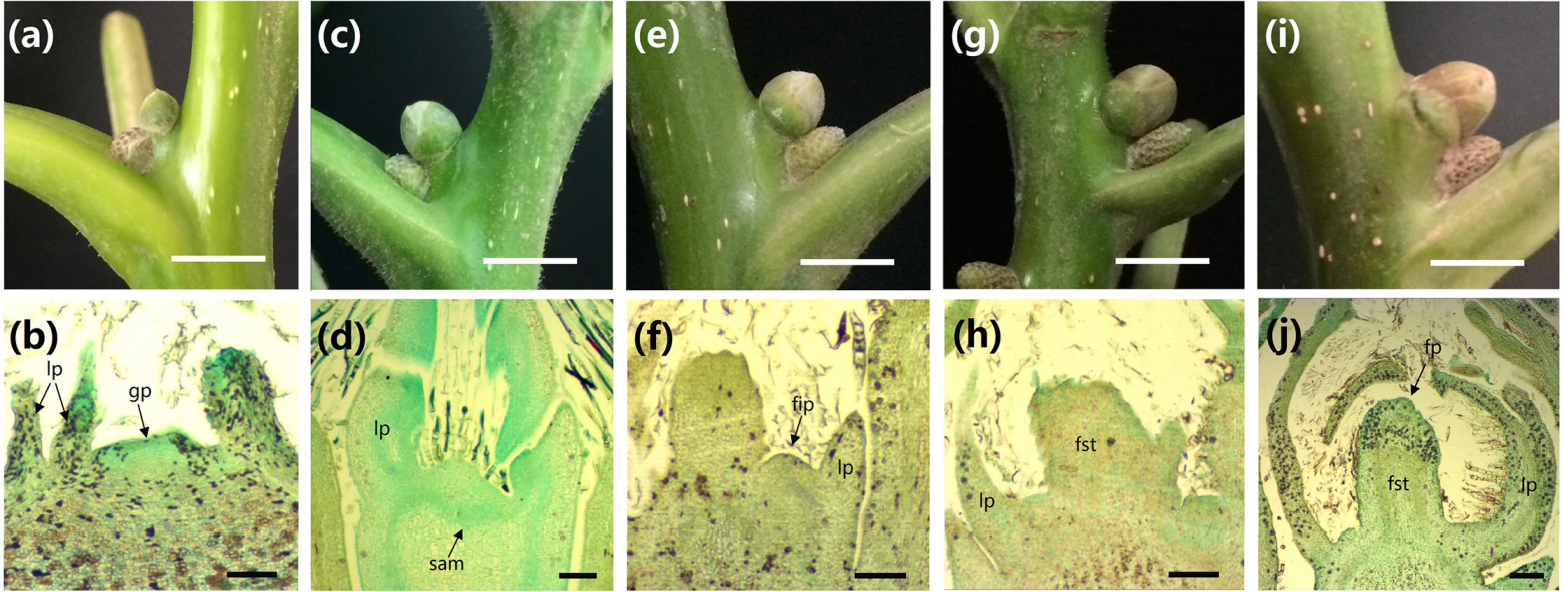
Morphological characteristics of the ‘Wen 185’ female flower bud. a, c, e, g and i present the phenotypic characteristics of five different developmental stages of female flower buds; b, d, f, h and j represent the internal morphological differentiation characteristics of five different developmental stages of female flower buds, a and b: The bud sprouted; c and d: Apical meristem began to flatten and sag; e and f: Spire turn rounded and shape protuberance; g and h: Flower stalk primordium development stage; i and j: Female flower primordium development stage. Ruler: 100μm. lp, leaf primordia; gp, growth point; sam, apical meristem; fip, female flower inflorescence primordium; fst, flower stalk; fp, female flower primordium.

In the Kashgar region of Xinjiang, the mixed buds of the ‘Wen185’ began to sprout in late March. After that, new branches began to grow, the axillary buds of the new shoot form and grow continuously (Fig 1A, 1C, 1E, 1G and 1I). While the external morphology of female flower bud changes, the internal development also continues. In P01, the internal growth of female flower buds was flat, and the leaf primordia already formed (Fig 1B); In P02, the apical meristem began to flatten and sag (Fig 1D). In P03, the growth point grows upward, and the spired turn round and shape protuberance (Fig 1F). In P04, the lower part of the globular bulge developed into the flower stalk primordium (Fig 1H). In P05, the top developed into the female flower primordium (Fig 1J).

### Full-length sequencing and acquisition of high-quality redundant sequences

To identify as many transcripts as possible, the concentration of RNA extracted from the female flowers of five different growth stages were mixed with equivalent content and used for library construction. Multiple size-fractionated libraries (1–2, 2–3 and 3–6 k) were constructed to avoid loading bias, which favoured sequencing of short transcripts (S2 Table). Eight cells were carried (four cells for 1–2 k libraries, two cells for 2–3 k libraries and two cells for 3–6 k libraries) (S2 Table). Filtered for sub-reads with length less than 50 bp and sequence accuracy less than 0.75, a total of 23.81 Gb of clean data containing 9,710,591 sub-reads were obtained (S2 Table). Each size-selected library had the expected distribution of mean transcript lengths ranging from 1,946 bp to 4,308 bp (S2 Table). Of the inserted reads, 240,258 of 616,540 were full-length reads based on the presence of bar-coded primers and polyA tails (S2 Table). The SMRT Analysis (v2.3.0) software using the ICE (Iterative Clustering for Error Correction) algorithm, combined with the quiver program, was used for sequence clustering. As a result, a total of 121,139 consensus isoforms were obtained, of which HQ (High-Quality) transcripts were 95,766 and LQ (Low-Quality) transcripts were 25,373. Transcriptomes sequence for the same samples were further used to correct the LQ transcripts (S3 Table). To get the non-redundant transcript sequences, HQ transcript sequences were mapped to the draft genome using GMAP (Genomic Mapping and Alignment Program). The sequences with identity less than 0.9 and coverage less than 0.85 were filtered. Reads differing only at the 5′-start site within the first exon was counted as redundant reads. Using this method, 52,875 non-redundant transcript sequences were obtained (S4 Table). Among of these non-redundant transcript sequences, 48,296 are similar to that of the known genome in transcript lengths. In which, a total of 6,951 transcript were completely mapped to the genome, but 41,345 transcripts with partial mapping. Additional 4,579 non-redundant transcripts were identified as the new transcripts. Thus, the walnut genome annotation was enriched with the SMRT results, and the integrated version was used for further analysis.

### Identification of novel genes, LncRNAs and alternative splicing events

**Fig 2:**
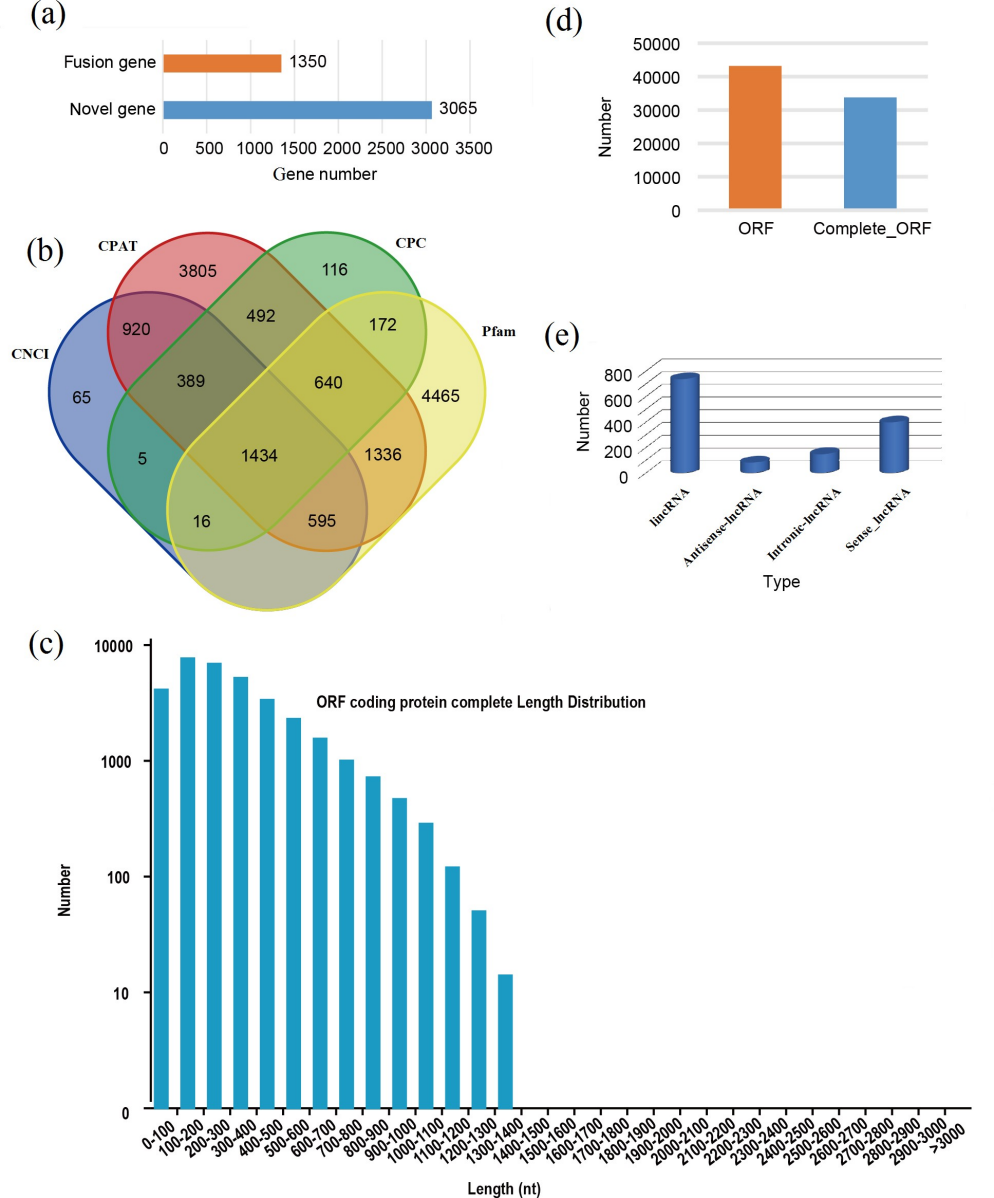
Identification of fusion genes, novel genes, lncRNAs, alternative splicing events and ORF numbers based on SMRT-based RNA sequencing. (a) numbers of fusion genes and novel genes, (b) Number of lncRNAs analyzed by pfam, cpat, cnci and cpc based on PacBio platform, (c) ORF coding protein complete length distribution, (d) numbers of ORF and complete ORF, (e) number and type of lncRNA. lncRNA, long non-coding RNA.

Prediction of novel genes, lncRNAs and AS events offers a new direction to further study the development of female flower buds in walnut. Using the transcripts detected by PacBio sequencing, 3,065 novel genes and 1,350 fusion genes were identified (Fig 2A). A total of 1,434 consensus lncRNAs were predicted by using CPC, CNCI, CPAT and PFAM software (Fig 2B). 14,313 lncRNA-mRNA pairs were predicted based on the location and LncTar software (S5 Table). In addition, a total of 43,171 ORFs containing 33,734 complete ORFs were obtained by using TransDecoder (v3.0.0) (Fig 2C and 2D).

Five kinds of alternative splicing (AS) patterns containing 20,813 AS events were identified based on Pacbio reads and corresponding annotated gene models (S6 Table). The number of alternative 3′ splice sites (17.61%) and retained introns (61.92%) were much more than alternative 5′ splice sites (9.21%), skipped exons (10.52%) and mutually exclusive exons (0.74%) (Fig 2E). Obviously, the majority of AS events is retained introns events.

### Transcriptome analysis of the flower buds in differently developmental stages

**Fig 3:**
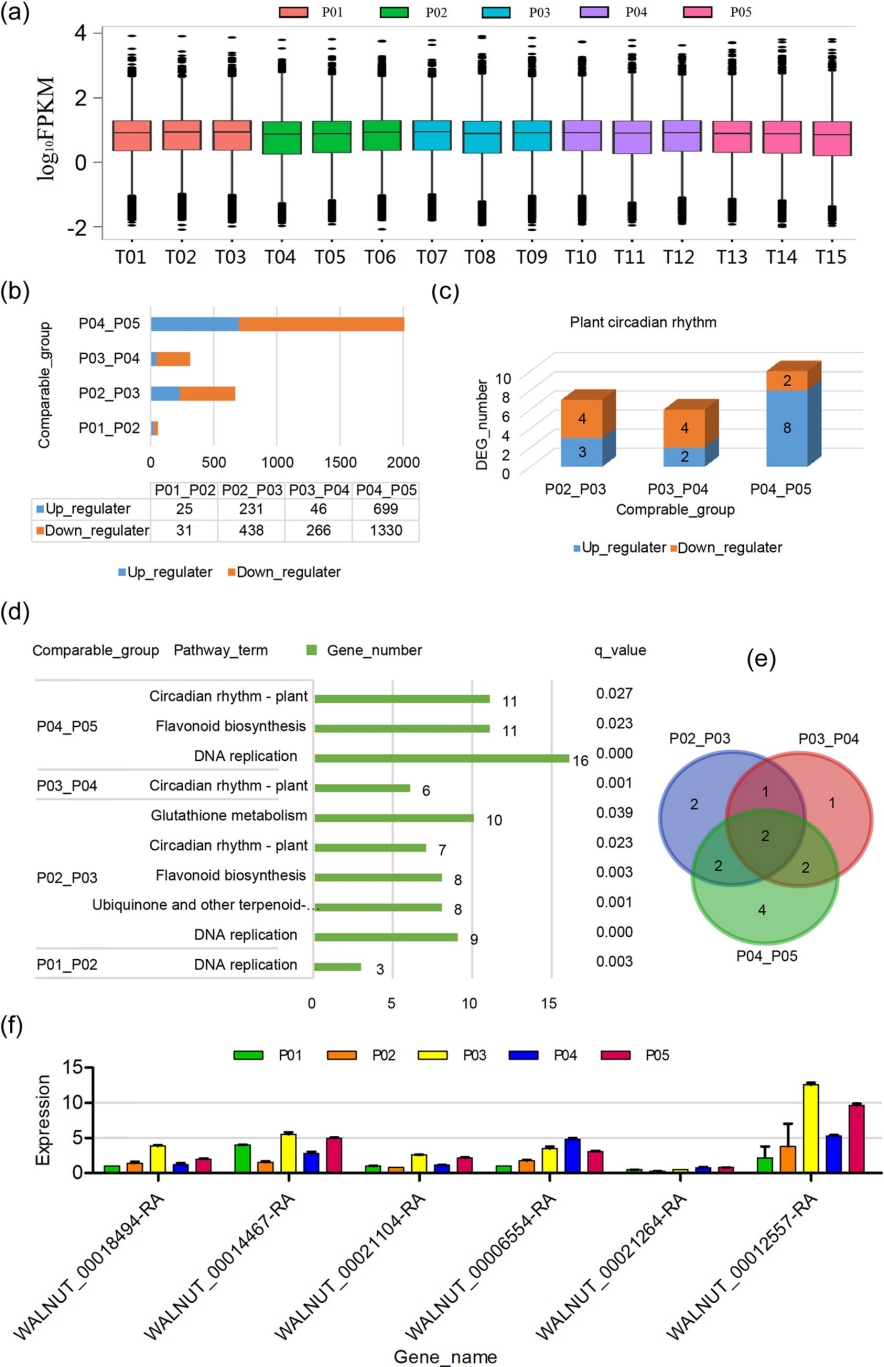
Identification of DEGs based on Illumina-based RNA sequencing. (a) Boxplots of transcript expression of female flower buds with three biological repeats at five different developmental periods, (b) number and regulated pattern of DEGs in different comparable groups, (c) KEGG enrichment analysis of DEGs in differently comparable groups, (d) number and regulated pattern of DEGs related to plant circadian rhythm in different comparable groups, (e) venn diagrams of DEGs related to plant circadian rhythm in differently comparable groups, (f) qRT-PCR analysis of selected plant circadian rhythm DEGs. DEG, differentially expressed gene. fpkm, fragments per kilobase per million mapped reads.

RNA-seq provided an overview of genes differentially expressed during different developmental stages. We constructed libraries and analyzed the sequences based on the Illumina HiSeq Xten platform. A total of 107.87sequencing data were obtained from the 15 libraries (P01, P02, P03, P04 and P05 with 3 replications), respectively (Table 1). After removing low-quality reads and adaptor sequences, 24.83, 21.74, 24.24, 23.94 and 25.50 million clean reads were generated, and more than 80% of the reads were uniquely mapped to the reference genome (Table 1). The transcript abundances of genes were estimated by fragments per kilobase of exon per million fragments mapped (FPKM). The boxplot distribution of the log_10_FPKM values in Fig 3A showed that the median and quartile values of the expression values across the libraries compared for differential expression were comparable.

**Table 1:** Summary of transcripts detected from RNA sequencing data.

Sample	Library	Base sum	Total reads	Uniquely mapped reads (%)	Reads mapped to multiple loci (%)	Reads mapped to many loci(%)	GC (%)	Q30(%)
P01	T01	7556617516	25298473	84.71%	7.50%	0.05%	45.91%	93.93%
	T02	7012081266	23464545	83.51%	7.00%	0.04%	45.51%	93.68%
	T03	7704480602	25754343	83.19%	6.88%	0.04%	45.55%	93.25%
P02	T04	6404233368	21456602	84.35%	7.37%	0.04%	45.39%	92.21%
	T05	5945529716	19913116	83.37%	8.25%	0.15%	45.6%	92.28%
	T06	7114499344	23859892	87.39%	8.09%	0.05%	45.59%	92.82%
P03	T07	7398788244	24743100	83.06%	7.10%	0.03%	45%	93.43%
	T08	6577597758	22011929	84.70%	7.85%	0.04%	45.75%	93.79%
	T09	7767310550	25980409	83.33%	7.07%	0.04%	45.22%	93.28%
P04	T10	6922008312	23166410	82.62%	7.53%	0.06%	45.32%	93.56%
	T11	7144878360	23865918	81.47%	7.03%	0.04%	45.39%	93.35%
	T12	7416817380	24788064	81.73%	7.28%	0.06%	45.25%	93.51%
P05	T13	7790691172	26016207	80.82%	7.55%	0.05%	45.6%	93.27%
	T14	7739820868	25875265	83.57%	7.18%	0.04%	45.13%	93.7%
	T15	7377869284	24632169	81.53%	6.86%	0.03%	45.31%	93.25%

To detect the DEGs related to the development of flower buds, we emphatically studied the gene expression patterns in the comparable groups: P01_P02, P02_P03, P03_P04 and P04_P05. Correspondingly, 56, 669, 312 and 2,029 DEGs were identified from P01_P02, P02_P03, P03_P04 and P04_P05, respectively (Fig 3B). The number of DEGs was least in P01_P02, but largest in P04_P05. The number of down-regulated genes were more than that of up-regulated genes in each comparable group. It can, therefore, be inferred that more and more genes were activated along with the progress of the flower development, and the negative regulatory genes are mainly dominanted during the flower bud development in walnut.

To explore the putative function of DEGs in the development of flower bud, KEGG pathway enrichment analysis was performed in each comparable group (Fig 3C). A total of 10 pathway terms contained 89 DEGs were significantly enriched (q <0.05) in the four comparable groups: one pathway contained three genes for P01_P02, five pathways contained 42 genes for P02_P03, one pathway contained six genes for P03_P04, and four pathways contained 38 genes for P04_P05, respectively. The pathway of DNA replication was significantly (*q* = 0.003) enriched in the P01_P02. P02_P03 and P04_P05. The flavonoid biosynthesis pathway was significantly enriched in the P02_P03 and P04_P05. The genes associated to DNA replication and flavonoid biosynthesis appear to especially express in the certain stage to affect flower bud development in walnut. Circadian rhythm-plant was commonly shared by P02_P03, P03_P04 and P04_P05. It implied that the circadian rhythm-plant pathway is the representation from the developmental stage P02, when was a initial stage of transformation of apical meristem to pistil primordium in walnut (Fig 1C and 1D). Thus, circadian rhythm-plant pathway was further investigated as candidate ones in the development of flower bud.

### DEGs associated with circadian rhythm-plant pathway

There were 14 DEGs to be annotated as circadian rhythm factors in three comparable groups (P02_P03, P03_P04 and P04_P05) (Fig 3D). Among of these DEGs, seven (three up-regulated genes and four down-regulated genes), six (two up-regulated genes and four down-regulated genes) and ten (eight up-regulated genes and two down-regulated genes) genes were identified in P02_P03, P03_P04 and P04_P05, respectively (Table 2). Two DEGs (*WALNUT_00003099-RA* and *WALNUT_00021104-RA*) were commonly detected in these three comparable groups. They were up-regulated in both P02_P03 and P04_P05, but were down-regulated in P03_P04. Similarly, *WALNUT_00021264-RA* showed differently regulated direction between P02_P03 and P03_P04. *WALNUT_00012557-RA* and *WALNUT_00018494-RA* exhibited the opposite trend between P03_P04 and P04_P05. These genes affected the development of flower bud by different regulated models along with the different flower bud developmental stages in walnut. Conversely, *WALNUT_00021631-RA* was down-regulated in both P02_P03 and P04_P05, but *WALNUT_00014467-RA* was up-regulated in these two comparable groups. It indicated that the expression of *WALNUT_00014467-RA* and *WALNUT_00021631-RA* might associate with the flower bud differentiation. *PB*.*6052* is first annotated as circadian rhythm factors by full-length sequencing, and positively regulated the development of flower buds from P04 to P05 (Table 2).

**Table 2:** List of DEGs related to circadian rhythm-plant pathway in walnut.

Walnut					*A*.*thaliana*		
Comparable_group	Gene_id	FDR	log_2_FC	Regulated	Homologous	Description	
P02_P03	WALNUT_00003099-RA	0.004	1.98	up	—		
	WALNUT_00014467-RA	0.002	1.81	up	*AT2G46790 (PRR9)*	Involved in clock function. Interact with TOC1 and act as transcriptional repressor of CCA1 and LHY.	
	WALNUT_00014989-RA	0.006	-7.40	down	*AT1G01060 (LHY)*	Encodes a myb-related putative transcription factor involved in circadian rhythm along with another myb transcription factor CCA1.	
	WALNUT_00016144-RA	0.007	-2.19	down	—		
	WALNUT_00021104-RA	0.000	1.40	up	*AT1G22770 (GI)*		
	WALNUT_00021264-RA	0.003	-8.24	down	—		
	WALNUT_00021631-RA	0.000	-2.41	down	—		
P03_P04	WALNUT_00003099-RA	0.002	-1.23	down	—		
	WALNUT_00006554-RA	0.000	1.29	up	—		
	WALNUT_00012557-RA	0.000	-1.32	down	—		
	WALNUT_00018494-RA	0.000	-1.88	down	*AT1G68050 (FKF1)*		
	WALNUT_00021104-RA	0.000	-1.57	down	*AT1G22770 (GI)*	Regulates several developmental processes, including photoperiod-mediated flowering, circadian clock.	
	WALNUT_00021264-RA	0.004	8.32	up	—		
P04_P05	PB.6052	0.001	Inf	up	—		
	WALNUT_00003099-RA	0.000	1.94	up	—		
	WALNUT_00008415-RA	0.000	1.02	up	*AT5G02810 (PRR7)*		
	WALNUT_00012557-RA	0.000	1.44	up	—		
	WALNUT_00014467-RA	0.001	1.25	up	*AT2G46790 (PRR9)*		
	WALNUT_00018494-RA	0.000	1.96	up	*AT1G68050 (FKF1)*	A flavin-binding kelch repeat F box protein, is clock-controlled, regulates transition to flowering.	
	WALNUT_00021104-RA	0.000	1.78	up	*AT1G22770 (GI)*		
	WALNUT_00021631-RA	0.002	-1.58	down	—		
	WALNUT_00024934-RA	0.008	-1.69	down	—		
	WALNUT_00026613-RA	0.000	1.21	up	*AT5G02810 (PRR7)*	Acts as transcriptional repressor of CCA1 and LHY.	

To further study the molecular function of the DEGs, they were used as query genes to detect their homologous genes in the model plant *Arabidopsis thaliana* (Table 2). *WALNUT_00014989-RA* is orthologous to *A*. *thaliana AT1G01060* (*LHY*), which was down-regulated in P02_P03. There are two genes *WALNUT_00008415-RA* and *WALNUT_00026613-RA*, which are both orthologous to *A*. *thaliana AT5G02810* (*PRR7*), up-regulated in P04_P05. Additionally, *WALNUT_00014467-RA*, *WALNUT_00018494-RA* and *WALNUT_00021104-RA* are orthologous to *A*. *thaliana AT2G46790* (*PRR9*), *AT1G68050* (*FKF1*) and *AT1G22770* (*GI*), respectively. To our knowledge, the *PRR7*, *PRR9*, *LHY*, *FKF1* and *GI* are extensively proved to be clock genes controlling the flower in *A*. *thaliana* [32–34].

To validate the results from RNA-seq data, we randomly selected six genes (*WALNUT_00014467-RA*, *WALNUT_00021104-RA*, *WALNUT_00021264-RA*, *WALNUT_00006554-RA*, *WALNUT_00012557-RA* and *WALNUT_00018494-RA*) from 14 circadian rhythm DEGs for real-time quantitative reverse transcription PCR (qRT-PCR) analysis. As expected, the expression profiles of these genes by qRT-PCR showed the similar trend with RNA-seq data (Fig 3F).

### Phylogeny and structure characteristics of the circadian genes

**Fig 4:**
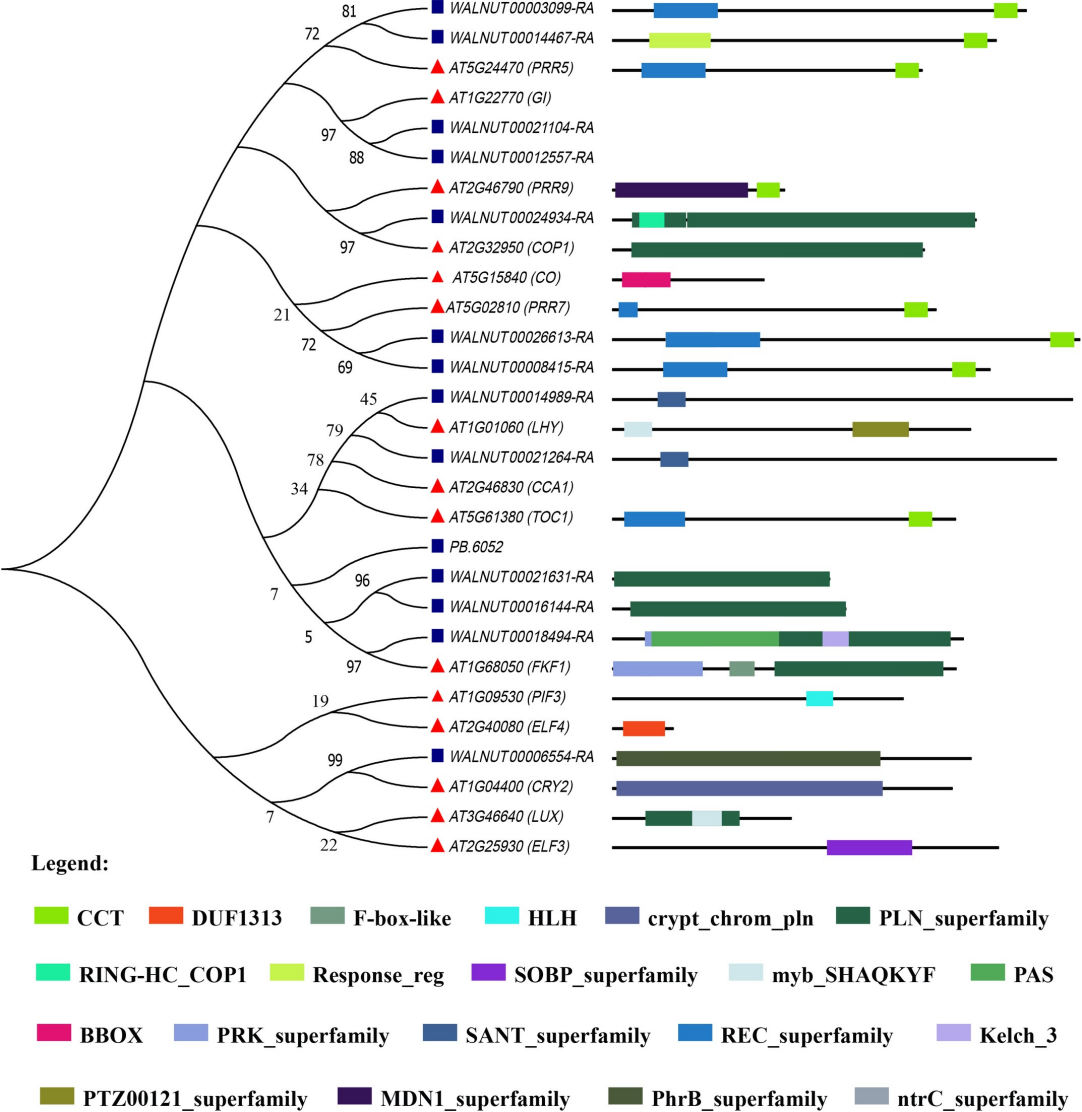
Phylogenetic tree and conserved structure domain of plant circadian rhythm proteins in walnut and *Arabidopsis*.

To gain insights into the phylogenetic relationship of the clock genes between walnut and *A*. *thaliana*, a phylogenetic tree was built by using their sequences (Fig 4). *WALNUT00021631-RA*, *WALNUT00016144-RA*, *WALNUT_00018494-RA* and their orthologous *FKF1* were grouped into the same cluster, they encoded the common PLN_superfamily domain. *WALNUT_00024934-RA* and *COP1* were grouped together, and they also encoded a common PLN_superfamily domain.

Proteins encoding by *WALNUT_00003099-RA*, *WALNUT00014467-RA* and *PRR5* shared common CCT domain, they were clustered into the same group. *WALNUT00026613-RA*, *WALNUT00008415-RA* combined with *PRR7* formed the same cluster, their proteins not only had a common REC_superfamily domain but also CCT domain. SANT_superfamily was both detected in *WALNUT00014989* and *WALNUT00021264*, which tended to form a same clay. *WALNUT_00012557-RA*, *WALNUT_00021104-RA* and their orthologous *GI* were clustered to the same clay. Similarly, *WALNUT_00006554-RA* and *CRY2* were grouped into the same cluster, their proteins had PhrB_superfamily and crypt_chrom_pln domain, respectively. Collectively, walnut circadian genes and their orthologous in *A*. *thalinia* tended to form the same clusters as their encoding proteins with same structure domain. These genes in the same clusters may act within the development of flower bud by displaying similar functions. Moreover, *PB*.*6052* formed a single clay without any predicted domain, implying that it may specially regulate the development of flower bud by response to circadian clock in walnut.

### AS events and lncRNAs coupled with expressed circadian genes

AS plays important role in mRNA processing [35,36]. Our results showed that eight expressed circadian genes were alternatively spliced with three types of splice isoform (Exon skipping, Intron retention, Alternative 3’ splice site) in walnut (S7 Table). Therein, intron retention (IR) was the predominant splicing pattern, with alternative 3′ splice sites ranking second and exon skipping ranking last. The proportion of different splicing events varied among DEGs. For *WALNUT_00012557-RA*, *WALNUT_00006554-RA* and *WALNUT_00003099-RA*, only a single splice isoform was detected, and others with more than two splice isoforms. All of these novel splice isoforms were newly annotated in the walnut genome.

LncRNA play a variety of biological roles in organisms [37]. In the study, large number of lncRNA-mRNA pairs were predicted based on full-length transcriptome sequences and transcriptome analysis. Among of these expressed circadian genes, *WALNUT_00003099-RA*, *WALNUT_00021104-RA* and *WALNUT_00012557-RA* were proved to be regulated by LncRNA.16269.3, LncRNA.18027.1 and LncRNA.18728.3, respectively (S7 Table). It implied the important roles of interaction between LncRNA and mRNA during the development of flower buds in walnut.

## Discussion

### Detection of novel genes, AS events and LncRNAs

*De novo* transcriptomes assemblies can be developed for the studies in functional genomics of plants. Mohammad Sadat-Hosseini [38] compared the assembled transcripts with transcripts of the walnut leaf, published genome assembly for the ‘Chandler’ cultivar using the BLAST algorithm and identified a number of novel homologue transcripts. This is a combine study of the full-length transcriptome sequences and transcriptome analysis to conduct the transcript detection of flower buds in walnut. Using this strategy, 52,875 non-redundant transcript sequences were obtained, and 4,579 new transcripts were identified with SMRT sequencing. Correspondingly, 3,065 novel and 1,350 fusion genes were identified by comparing with the walnut genome [16]. Among of these novel genes, *PB*.*6052* was annotated to response to circadian rhythm-plant, and whose expression was associated with the development of flower buds in walnut. Which is an excellent complement to the single genome in walnut. On the one hand, walnut exhibits a strong reduction in genetic variation due to population bottlenecks and strong artificial selection during domestication, which means certain functionally important genes lost in the reference walnut genome. On the other hand, characterizations of the reference walnut genome rely on short reads sequencing, and the genome gap regions may reduce the gene annotation accuracy. A total of 1,437 consensus lncRNAs and 14,313 lncRNA-mRNA pairs were predicted based on SMRT sequencing. Additionally, 20,813 AS events were first found in walnut genome based on Pacbio reads and corresponding annotated gene models. The results indicated that large-scale full-length cDNA sequence based on SMRT is a powerful tool for identifying novel genes/isoforms, which has greatly improved the current genome annotation and enhanced our understanding of the walnut transcriptome.

### Detection of circadian genes for the flower bud development

Circadian clocks fine-tune physiology and behaviour to match the environmental day/night cycle [39,40]. The plant circadian clock generates daily and even seasonal rhythms in an orderly succession of physiological processes including stomatal opening, leaf movement, hypocotyl elongation, photosynthesis and carbon metabolism, resistance to abiotic and biotic stresses, and flowering time [15,33,41–45]. Expression analysis of critical genes helped to dissect the genetic basis of important traits, such as floral in hickory [46] and Persian walnut [47]. Based on our analysis, the pathway of circadian rhythm-plant was enriched during the development of flower buds of walnut, which is also observed by Quan [15]. A total of 14 DEGs involved to the circadian rhythm were identified during the study. Homologous comparison and phylogenetic analysis revealed that *JrFKF1*, *JrGI*, *JrPRR5*, *JrLHY*, *JrPRR9* and *JrPRR7* are highly orthologous to the corresponding genes in *Arabidopsis thaliana*, indicating their similar functions in these two species. Additionally, *JrCOP1-like*, *CRY2-like*, *JrPRR5-like*, *JrFKF1-like*, *JrLHY-like* and *JrGI-like* were first reported to confer flower bud development in walnut. All these DEGs provided a valuable resource for further gene cloning.

### Regulatory network of circadian genes

**Fig 5:**
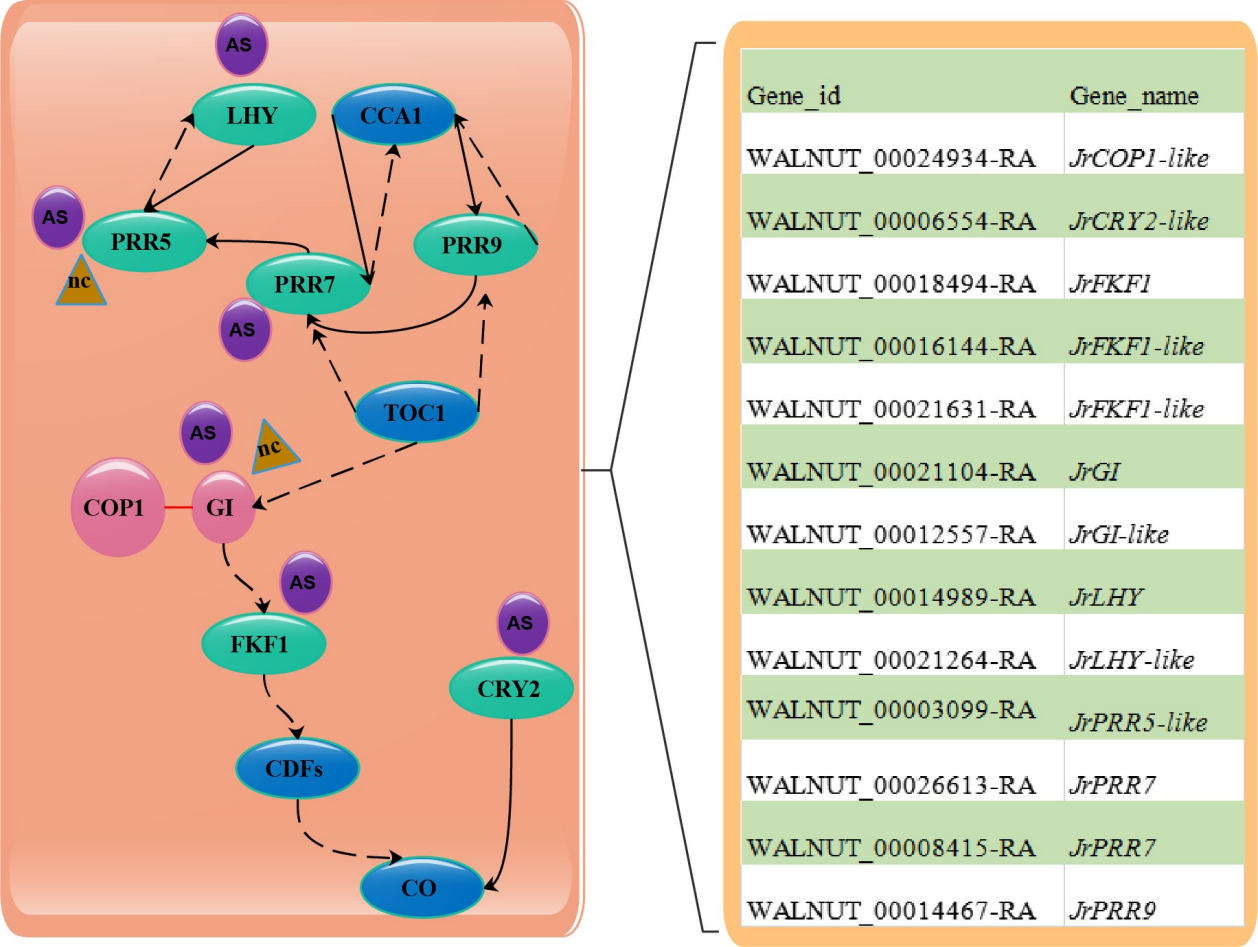
Hypothetical model for plant circadian rhythm genes regulating flower development in walnut. AS, alternatively splice; nc, long non-coding RNAs.

Extensively excellent reviews and references had reported the details about the clock machinery [33,44]. The morning-expressed MYB-like transcription factors *CCA1* and *LHY* and the evening expressed *TOC1* reciprocally repress each other’s expression, which produces a feedback loop forming the core of the clock machinery. *CCA1* and *LHY* also act as activators and repressors of the dayphased clock genes *PRR9*, *PRR7* and *PRR5*. Additionally, *TOC1* have been shown to repress several other evening genes, such as *GI* (Fig 5). In the study, *JrLHYs* (*WALNUT_00014989-RA* and *WALNUT_00021264-RA*) showed a contrasting expression pattern to *JrPRRs* (*WALNUT_00014467-RA*, *WALNUT_00003099-RA*, *WALNUT_00026613-RA* and *WALNUT_00008415-RA*) from P02 to P05 (Fig 5). Similar results were also observed for *JrGIs* (*WALNUT_00021104-RA* and *WALNUT_00012557-RA*) and *JrFKF1s* (*WALNUT_00016144-RA* and *WALNUT_00021631-RA*), the repression effect was also detected in walnut by Quan [15]. These results indicated that the repressions of these clock factors mainly affect the flower bud development in walnut.

As far as we know, *FKF1* is F-box protein acted as blue-light receptor to regulate *CO* expression [48]. GI is nuclear protein physically interacted with diverse proteins acted under short period [49]. The interaction of *JrGI* and *JrCOP1* was previously found during the floral transition in walnut [15]. In this context, *JrGI* was up-regulated, while *JrCOP1-like* (*WALNUT_00024934-RA*) was down-regulated from P04 to P05, indicating their opposite regulation pattern. We also found that *JrGI-like* (*WALNUT_00012557-RA*) shared the same regulation direction with *JrFKF1* (*WALNUT_00018494-RA*) from P03 to P05 (Fig 5). Their interactions may play important roles in regulating the growth point eventually forms the flower primordium in walnut.

*CRY2* was functioned as blue-light receptors to activates *CO*, thereby driving the expression of *FT* [50]. In the result, *JrCRY2-like* was only detected to up-regulate from P03 to P04. Similarly, merely up-regulated the novel gene *PB*.*6052* contributed to the flower transition from P03 to P04 (Fig 5). These genes play a role in regulating the formation of flower stalk primordium as showed for paraffin section in walnut (Fig 1F and 1H).

### Regulation of circadian genes expression by AS and LncRNAs

AS and lncRNA are widespread in plant, and have been proved to be involved in many biological processes [37,51,52]. To date, however, there is little knowledge about the AS and lncRNAs events, especially their effects on circadian genes in walnut. Herein, transcriptome analysis revealed that AS events occurred for *JrPRR5*, *JrPRR7*, *JrLHY*, *JrGI* and *JrCRY2*. The observation of AS events for *LHY*, *PRR5* and *PRR7* were also reviewed by James [53]. Among of the AS events, IR was the predominant splicing pattern, which was partly consist with the observation that IR is the most frequent kind of AS in flowering plants [36]. A suite of lncRNAs have been identified to function in reproductive organs in rice [54] and maize [55]. Wang [56] recently identified lots of microRNAs differentially expressed involved in male flower development in hickory. Fan [57] provides a genome-wide survey of hickory flower-related lncRNAs and studied the roles of lncRNA underlying the molecular mechanism underpinning flowering in hickory. Fan [58] verified that a class of long non-coding RNAs mark enhancers modulating long-range circadian gene regulation in mouse. In this context, another class of lncRNAs were first predicted to regulate clock genes (*JrPRR5* and *JrGI*) in walnut (Fig 5). It is expected that the results contribute to the understand the roles of lncRNA and AS in the development of flower buds in walnut.

## Conclusion

The full-length transcripts using PacBio Iso-Seq can greatly improve the genome annotation and broaden our perspectives of the transcriptome features in walnut. On the other hand, RNA-seq analysis helped identifying 14 DEGs functioning in a manner of potentially regulate the flower buds developmental by acting the circadian rhythm-plant in walnut, indicating than investigation of key genes associated with the circadian clock could clarify the process of flower bud developmental in walnut. Finally, the detected AS events and lncRNAs provide serviceable regulator data pools for further screening and functional verification. This paper provides a foundation for further research on the walnut floral development.

## Supporting information

S1 Table Primer sequences used for quantitative real-time polymerase chain reaction (qRT-PCR) expression analysis. (XLSX)

S2 Table Statistics of full-length sequencing data based on PacBio. (XLSX)

S3 Table Statistics of inserted reads for full-length sequencing based on PacBio. (XLSX)

S4 Table Filtration of non-redundant transcripts sequenced by full-length sequencing based on PacBio. (XLSX)

S5 Table Identification of long non-coding RNAs sequenced by full-length sequencing based on PacBio. (XLSX)

S6 Table Identification of AS events sequenced by full-length sequencing based on PacBio. (XLSX)

S7 Table AS events and lncRNAs coupled with expressed circadian genes. (XLSX)
